# Burnout, Compassion Fatigue and Psychological Flexibility among Geriatric Nurses: A Multicenter Study in Spain

**DOI:** 10.3390/ijerph18147560

**Published:** 2021-07-15

**Authors:** Carmen Sarabia-Cobo, Victoria Pérez, Pablo de Lorena, Ángela Fernández-Rodríguez, José Rafael González-López, Julia González-Vaca

**Affiliations:** 1 Facultad de Enfermería, IDIVAL, Universidad de Cantabria, Avda Valdecilla s/n, 39011 Cantabria, Spain; angela.fernandezr@unican.es; 2 CR Santa Lucía, 28001 Madrid, Spain; vperrez.had@gmail.com (V.P.); pdelorena.had@outlook.es (P.d.L.); 3 Department of Nursing, Faculty of Nursing, Physiotherapy and Podiatry, Universidad de Sevilla, C/Avenzoar nº6, 41009 Seville, Spain; joserafael@us.es; 4 Departament D’Ínfermeria Medicoquirurgica, Campus Bellvitge, Universidad de Barcelona, 08907 Barcelona, Spain; juliagonzalezvaca@ub.edu

**Keywords:** nurses, stress, psychological, compassion fatigue, mental health, geriatric nursing

## Abstract

Nurses working at nursing homes are one of the most vulnerable populations for suffering burnout and compassion fatigue. In Spain, the concept of compassion fatigue and psychological flexibility related to stress in geriatric nurses has not been fully explored until now. It is important to analyze their situation in order to design robust coping and management strategies. The aim was to analyze the relationship between burnout, compassion fatigue and psychological flexibility in geriatric nurses in Spain. Participants included 291 nurses from 97 centers in 51 cities across Spain. Psychological flexibility (AAQ-II), burnout (MBI) and compassion fatigue (ProQOL) were evaluated. Responses were recievced from 281 nurses (91% women), with an average of 7.6 years of work experience. The MBI results were average (26.71), and the ProQOL scores were average for compassion fatigue (40.2%) and high for compassion satisfaction (70.3%), whereas for AAQ-II, the mean score was 37.34 (SD 4.21). The correlation was significant and negative for flexibility, burnout and compassion fatigue, and positive for compassion satisfaction. The ANOVA indicated a significant association between all variables (*p* < 0.05). We can conclude that geriatric nurses suffer from medium levels of burnout and compassion fatigue, together with high levels of psychological flexibility, which appears to act as a stress reliever, supporting compassion satisfaction.

## 1. Introduction

As early as 1970, Louis Davis underlined the need for organizations to be concerned about the welfare and health of all employees, to support optimal performance of duties [1]. Currently, this encompasses aspects related to the physical, environmental and psychological aspects of the workplace [2]. 

In the field of healthcare, the intricacy of care work (work shifts, relationships with patients and relatives, direct contact with illness, pain and death, lack of professional recognition) means that healthcare professionals suffer from chronic stress, which leads to a high incidence of burnout, as shown in literature reviews, such as a report by Dreison et al., [3] analyzing findings over the last 35 years, or a study by Hall which analyzed forty-six studies [4]. In geriatrics, these factors are even more pressing [5]. Health professionals specializing in this field are under great pressure. They must establish links with families to gain their support and trust, and have to deal with elderly people with emotional and behavioral disorders. This is especially true in dementia, which is characterized by elevated care needs [6]. As dementia is a long-term neurodegenerative disease, both professionals and caregivers are at risk of being subjected to chronic stress and high demand, as corroborated by the literature [7]. Over time, all of these drawbacks can de-motivate the professional, increasing work stress, favoring the appearance of burnout, deteriorating the quality of life and, inevitably, also the quality of care [8,9]. It is important to remember that burnout is understood as the psychological discomfort that is manifested as decreased performance at work, as a result of maladaptive attitudes and behaviors, representing a strong predictor of work dissatisfaction, and directly related to the development of health problems [10,11]. Burnout implies inadequate patient care, risks to patient safety and a decline in the quality of care [2,3,4,5,6,7,8,9,10,11,12,13,14]. 

According to Buunk and Schaufeli [15] caring for people who are suffering comes with a high personal cost, as it can lead to a decline in work, social and family functions. Sometimes, professionals may feel that their sense of self is lost. In addition, Figley [16] emphasizes the importance of knowing that the ability to develop empathy is one of the keys for working well with people who suffer, however, at the same time, empathy can generate an emotional imbalance due to burnout and psychological fatigue, known as compassion fatigue or empathy fatigue. The concept of empathy fatigue (also called compassion fatigue) is a term that was born in 1995 at the hand of Charles Figley, coined as “compassion fatigue”. For Charles Figley [16], empathy attrition or compassion fatigue is a concept that relates to the cost of care. According to the Webster Encyclopedic Unabridged Dictionary of the English Language (cited by Figley) [17], empathy fatigue is “a feeling of deep empathy and sorrow for someone who is suffering”. The difference between a healthy professional who works with human suffering and one who has the syndrome is not that both do not suffer the symptoms of empathic burnout, but rather the chronic nature of the symptoms, as pointed out in recent literature reviews [18,19,20,21]. Although it is a concept that was born several years ago in Spain, its meaning has been poorly studied and is often confused with other terms, including the much more studied burnout syndrome [22,23]. 

Potter et al., [24] state that one of the differences between empathy fatigue and burnout is that the former can arise in a single session, unlike the latter, which is a process. In contrast, empathy fatigue is isolated, and even immediate. Another difference stated by Figley [17] is that, among other consequences, burnout can result in a change of work or career. However, compassion fatigue is highly treatable once professionals recognize it and take steps to address it [25]. Showalter [26] explains that another notable difference between burnout and empathy fatigue is that, although both produce a state of physical, mental and emotional exhaustion produced by being involved in situations of high emotional demand over a long period of time, burnout arises independently of the empathy being provided and without the need for specific exposure to a user’s suffering.

The literature indicates that nurses are at high risk of developing compassion fatigue and stress [27,28]. Studies indicate that improved education and training can have a buffering effect on compassion fatigue and stress and may improve nurses’ mental health and thus the quality of care provided [29,30]. Currently, much research is being conducted on the application of Acceptance and Commitment Therapy (ACT) [31], which aims to increase valuable personal behaviors, increasing the recognition and acceptance of personal experiences associated with discomfort (thoughts, emotions and feelings) and what this entails: letting go of control as a strategy that increases the potential for discomfort [32]. An important concept that is addressed in this therapy and is closely related to stress is psychological flexibility, which has been defined as the “ability to fully contact with the present moment and the thoughts and feelings it contains, without the need for defense, and, depending on the situation, to change or persist in the behavior, in order to achieve valuable goals” [33]. 

Studies have shown relationships between psychological flexibility and important workplace behaviors. Higher levels of psychological flexibility appear to predict better mental health and job performance [34]. Psychological flexibility, although stable over time, is an individual characteristic that can be improved, and research has shown that it can, in turn, improve work-related behavior; for example, controlled trials show that an increase in psychological flexibility is the mechanism by which ACT interventions improve overall mental health [35,36,37].

In Spain, the concept of compassion fatigue and psychological flexibility related to stress in geriatric nurses has not been fully explored until now, unlike other international settings [38,39,40]. 

Considering that nurses working at nursing homes are one of the most vulnerable populations for suffering burnout and compassion fatigue, it is important to analyze their situation in order to design robust coping and management strategies, as suggested in the literature [41]. 

Therefore, the aim of this study was to analyze the relationship between burnout, compassion fatigue and psychological flexibility in geriatric nurses who work in nursing homes, via a multicenter study in Spain.

## 2. Materials and Methods

A cross-sectional online survey was designed and hosted using the SurveyMonkey platform. We followed the Transparent Reporting of Evaluations with Nonrandomized Designs (TREND). The data collection period took place during January and February 2020.

### 2.1. Sample/Participants

The questionnaire was sent to 97 care centers for older people belonging to the same religious community, distributed among 51 Spanish cities throughout the country. In total, 291 nurses received the questionnaire. The inclusion criteria were: at least six months of experience in the current job and not being on sick leave due to depression, psychiatric disorder or anxiety.

### 2.2. Variables and Instruments

- Independent variables: age, gender, number of years working at the center, marital status, work shift.
- Dependent variables:

Psychological flexibility was measured using the Acceptance and Action Questionnaire II (AAQ-II) [42] in its version adapted to Spain [43]. This questionnaire is based on a 7-point Likert scale. The total scores range from 7 to 49. In alignment with other processes in this study, this scale was scored inversely, whereby higher scores indicated higher levels of psychological acceptance and flexibility (i.e., less experiential avoidance).

Burnout was measured using the Maslach Burnout Inventory MBI Scale. This consists of 22 items that are valued on a Likert scale (six possible responses ranging from “never” to “daily”) to determine the frequency with which a series of situations are experienced. Three dimensions are evaluated, organized into three subscales: emotional fatigue, depersonalization and professional fulfillment.

Compassion fatigue was assessed using the Professional Quality of Life Scale (ProQOL) [44,45]. This scale measures the risk of compassion fatigue and risk of burnout as negative consequences of helping professions, and the level of compassion satisfaction experienced by the assessed subject. These three dimensions are evaluated based on 10 items for each dimension, which are scored on a 6-point Likert-type scale ranging from 0 (Never) to 5 (Always).

### 2.3. Data Collection

The study was announced to potential participants in all centers through e-mail systems and the intranet. The link to the questionnaire was provided to all interested persons by e-mail. They were also provided with information about the content of the study and were asked to complete the study in a single session, whenever they wished. All participants gave their consent before opening and completing the survey. On average, participants completed the questionnaire (including instructional reading time) in 12 min (SD 3.12). Participants’ responses were exported to the IBM SPSS v.25 database and prepared for data analysis.

### 2.4. Ethical Considerations

Authorization was obtained from the Foundation that managed all the centers, as well as from the centers themselves. In addition, the Bioethics Committee of the managing Foundation granted ethical permission for this study (EC 15/2019). All data were anonymous and were treated according to Spain’s current legislation. All participants gave written consent to participate in the study.

### 2.5. Data Analysis

IBM SPSS Statistics 25 software was used for statistical analysis. A bilateral contrast and a 95% confidence level were adopted. A descriptive analysis of all the variables collected was performed for each group. Parametric assumptions (linearity, homoscedasticity and outliers) were visually inspected, and the normality of the distribution was verified. Multifactorial ANOVA and the Pearson’s correlation were used to identify potential covariates. Linear regression was used to analyze the relationship between psychological flexibility (as an outcome variable) and the remaining variables (as covariates) using backward elimination. Statistical significance was set at *p* < 0.05.

### 2.6. Validity and Reliability/Rigor

The study design ensures the criteria of validity and rigor: validated and reliable instruments and tests have been used, with good psychometric properties of internal consistency and validity, appropriate and adapted to the study population. Likewise, the design of data collection and its subsequent statistical analysis have followed the relevant quality and robustness criteria that allow for obtaining reliable, replicable and generalizable results.

## 3. Results

Of the 291 nurses who were sent the link to the questionnaire, 281 responded to the questionnaire (96.6% response rate). Table 1 shows the main socio-demographic variables.

Regarding the findings for burnout, analyzing the MBI scale for all professionals (*n* = 281) the mean score was 26.71 (SD 7.23). In the subscale for emotional exhaustion, we found that 37.2% presented high levels and 47.6% presented medium levels of emotional exhaustion, in the subscale for depersonalization, 21.8% presented high levels and 59.4% presented low levels of the same, and for the subscale of performance at work, 26.6% presented high levels, 38.6% presented low levels and 34.8% presented medium levels. Younger people scored higher on emotional exhaustion and depersonalization, although there were no statistically significant differences (t = 12.34, *p* = 0.08; *p* = 0.07). There were no major differences between the three subscales by employment status, gender or work shift. We explored if there were differences between men and women on psychological flexibility and if gender moderates its relationship with burnout and depression, but there was no significant result.

In terms of findings for compassion fatigue, according to ProQoL, scores below 22 indicate low levels, 23–41 are average and those above 42 are considered high for each subscale. Within our sample, burnout and compassion fatigue levels were in the middle range (burnout: low = 24.5%, average = 75%, high = 0.5%; compassion fatigue: low = 59.8%, average = 40.2%). The scores for levels of compassion satisfaction were comparatively higher within our sample (low = 1.4%, average = 70.3%, high = 28.3%). Statistically significant differences were found for compassion fatigue by years worked, with these percentages being higher with more years of work experience (t = 9.25, *p* = 0.02). No statistically significant differences were found for compassion fatigue and compassion satisfaction by age, gender, professional category or shift.

In terms of psychological flexibility, measured using the AAQ-II, the mean score was 37.34 (SD 4.21), which is considered a high range, with statistically significant differences according to age: the older the person, the more flexibility (t = 24.14, *p* = 0.00), additionally, the more years worked, which also indicates more years of work experience, the greater the flexibility (t = 32.74, *p* = 0.00). The results of the correlation study between variables are shown in Table 2.

This study has shown that high levels of burnout are positively correlated with presenting compassion fatigue and inversely correlated with psychological flexibility and compassion satisfaction. We also observed a significant negative correlation between compassion fatigue and compassion satisfaction and flexibility. Likewise, the latter correlates positively and significantly with compassion satisfaction.

A multifactorial ANOVA, considering psychological flexibility as an independent variable and burnout, compassion fatigue and compassion satisfaction as dependent variables, indicated a statistically significant relationship between flexibility and burnout (F(8, 261) = 12.34, *p* = 0.02), between flexibility and compassion fatigue (F(8, 261)= 23.54, *p* = 0.03) and between flexibility and compassion satisfaction (F(8, 261)= 9.45, *p* = 0.01). A multiple regression analysis (MRA) with the forward stepwise method was used to investigate potentially predictive factors for the development of psychological flexibility. The final coefficients are presented in Table 3.

All variables were entered into the regression model at the first step.

## 4. Discussion

The aim of this study was to analyze the relationship between the psychological variable flexibility with variables that have a major impact on the mental health of nurses, such as compassion fatigue and burnout. Our findings revealed that in our sample of 281 nurses working in direct care of older people in long-term care facilities, the ranges of burnout and compassion fatigue were medium, whereas the ranges of compassion satisfaction and flexibility were high. We also found a significant association between flexibility as a protective variable compared to compassion fatigue and burnout. Compared to other results, the range of burnout and compassion fatigue is slightly higher for the values studied in palliative care nurses, critical care units and others, and is generally at medium levels [6,27,46,47,48]. 

Likewise, recent systematic reviews and meta-analyses show that compassion fatigue is a phenomenon that has been studied more recently, in comparison with other phenomena such as burnout. This highlights the importance of the psycho-emotional and behavioral consequences that can arise from continued exposure to experiencing the suffering and pain of people who nurses care for during their illness [3,22,49]. 

There are many studies on the medium and high levels of burnout in these groups. Burnout involves negative attitudes and behaviors in good job performance in nursing in response to work stress [50]. It is common for healthcare professionals who must care for complex patients who require extensive care (such as the elderly with dementia) to experience reduced compassion satisfaction, increased compassion fatigue and burnout. Therefore, nurses suffer from more emotional burnout than other healthcare professionals [51]. It seems clear that the characteristics of the job, lack of time and the pressure to attend to patients, generate an impact on the mental health of nurses, which, if not properly managed, leads to chronic stress [28]. These facts, together with the professional’s own personal variables, have an impact on the appearance of burnout and compassion fatigue, as indicated by a recent systematic review in the USA, in which twenty primary source publications between the years 2010 and 2017 were analyzed, reporting that the factors for compassion fatigue and burnout were age, years working as a nurse, work environment, coping mechanisms and specialties [52]. 

Our results seem justified and aligned with the reality of care provided in the geriatric environment of nursing homes, where burnout and compassion fatigue outcomes are higher than in other care settings [7,40,53]. Work in long-term care facilities involves the complex care of patients and families that can lead to stress and emotional strain. Emotional stress may be due to repeated exposure to suffering, failed attempts to alleviate such suffering, frequent deaths and the conflict that may arise between the “cure” vs. “care” paradigm, especially in the context of older people [54,55]. This could be so because through empathy, nurses can come to internalize and become engrossed in the problems of the people they help [56,57]. Figley also adds another cause to justify the greater vulnerability of this group, considering that compassion and empathy are fundamental and indispensable values in this profession [17]. 

Moreover, according to our results, it is interesting to note that older and more experienced nurses had significantly higher rates of flexibility and compassion satisfaction than younger and less experienced nurses. This is corroborated in other studies, indicating that experience and maturity over the years increases psychological flexibility and the capacity for acceptance developed in adverse situations [29]. In this sense, it is important to continue investigating what resources the worker can use to help maintain high states of energy and motivation on and off the job, throughout the years of working life. Thus, the study of psychological flexibility and full attention as personal resources in the field of nursing can be very interesting in light of the studies that have recently appeared in the literature and that relate both to the levels of professional burnout and work engagement within this collective.

It seems clear that psychological flexibility acts as an effective modulator for the control of burnout and compassion fatigue, while at the same time enhancing compassion satisfaction as a protective variable [58]. Therefore, in recent years, we have seen an increase in studies aimed at improving flexibility and acceptance through interventions based on the theory of acceptance and commitment, with very good results [32,35,59]. Indeed, interventions aimed at increasing psychological flexibility may even prevent the development of burnout syndrome [36,37]. In this sense, the literature indicates that workers with greater psychological flexibility do not engage in constant avoidance strategies and accept their emotions as part of the path they have decided to take, which is directed towards their goals and values.

As a result, they are able to follow through and respond more effectively to opportunities related to their goals [60]. This is because the professional’s attention and energy are not directed at controlling negative internal experiences, and therefore, they are more sensitive to the information provided by the work environment to initiate goal-directed behaviors [61]. In addition, coping based on psychological acceptance is less resource-intensive than coping based on emotional control [62]. Thus, psychological flexibility has also been shown to be associated with increased performance and productivity [60] increased vitality [63] and decreased levels of emotional burnout [56]. All of this seems justified as, in our study, high levels of psychological flexibility were positively associated with compassion satisfaction and negatively associated with compassion fatigue and burnout. Evidence-based interventions that promote compassion satisfaction and address negative stress outcomes are essential to equip nurses with stress management skills to promote their professional quality of life and mental health [42,56].

### Limitations

Finally, this study has some limitations that are worth noting. Due to the cross-sectional nature of the study, it is impossible to establish causal relationships between the variables, considering that we used a convenience sample. Furthermore, although all the centers work in the same line, there is also a location bias, as each center has its own idiosyncrasy and work environment, and therefore the results may not be as homogeneous. Our study strengths are the large sample size, and the fact that this was a nationwide multicenter study, being the first of this type carried out in Spain, focused exclusively on the area of nursing in old people’s homes.

Future lines of research should offer professionals the possibility of increasing their levels of psychological flexibility and mindfulness through standardized training programs that have shown their effectiveness in the healthcare organization, such as stress reduction interventions based on Acceptance and Commitment Therapy and mindfulness. Another interesting line of research is to carry out longitudinal studies to observe the value and relationship between the study variables over time. It seems clear that an interesting line of research will be to reassess all these parameters during the COVID-19 pandemic we are currently experiencing.

## 5. Conclusions

In light of our results, we can conclude that emotional exhaustion at work is a personal state in which contextual and personal factors interact, and where psychological flexibility seems to be a protective element against burnout and compassion fatigue, which are intermingled and overlapping in professionals, making it difficult to detect and act upon such factors. Nurses who care for older people, who are in the final stage of life, are exposed to greater emotional distress, negatively affecting their mental health, compared to other specialties. Solutions to increase psychological flexibility and to alleviate high levels of burnout and compassion fatigue should begin with early detection to enable appropriate interventions and to prevent a negative progression. To act upon these problems, it is necessary for strategies to be articulated, stemming from the affected person, their colleagues and the organization where they work. Preventive measures and mental health programs for workers are a factor that must be considered, due to the costs that can be saved on an economic and human level. It is necessary to combine the implementation of certain training programs and psychological interventions with healthcare staff.

## Figures and Tables

**Table 1:** Sociodemographic variables.

	Nurses (*n* = 281)
Age (M/SD)	36.8 (5.4)
Gender	91% women
Work situation	
Intern	11.8%
Permanent employee	72.8%
Temporary	15.4%
Years of work experience (M/SD)	7.6 (4.5)
Shift	
Fixed	11%
Rotational	47%
Split-shift	42%

**Table 2:** Correlational study between psychological flexibility, burnout and compassion fatigue, and compassion satisfaction variables.

	Mean (SD)	1	2	3	4
1. Burnout	26.71 (7.23)	–			
2. Compassion fatigue	22.85 (2.35)	0.74 *	–	–	–
3. Compassion satisfaction	38.45 (8.41)	−0.65 *	−0.71 *	–	–
4. Psychological flexibility	37.34 (4.21)	−0.74 *	−0.78 *	−0.58 *	–
5. Age	36.8 (5.4)	0.47	0.65	0.88	0.87 *
6. Years of work experience	7.6 (4.5)	0.58	0.74	0.25	0.74 *

* = *p* < 0.001.

**Table 3:** Multiple regression analysis (MRA).

Model	*B*	β	*t*	*p*
Burnout	51.62	–	27.13	<0.001
Compassion fatigue	42.12	–	19.48	<0.001
Compassion satisfaction	46.71	–	18.31	<0.001

All variables were entered into the regression model at the first step.

## Data Availability

Not applicable.
